# Malignant transformation of oral epithelial dysplasia in Southwest Finland

**DOI:** 10.1038/s41598-022-12441-9

**Published:** 2022-05-18

**Authors:** Toni T. Nevanpää, Antti E. Terävä, Hanna K. Laine, Jaana Rautava

**Affiliations:** 1 Oral Pathology and Oral Radiology, Faculty of Medicine, University of Turku, Turku, Finland; 2 Department of Oral and Maxillofacial Diseases, Clinicum, Faculty of Medicine, University of Helsinki and Helsinki University Hospital, P.O. Box 41, 00014 Helsinki, Finland; 3 Department of Pathology, Medicum, Faculty of Medicine, University of Helsinki and HUS Diagnostic Center, HUSLAB, Helsinki University Hospital, Helsinki, Finland

**Keywords:** Cancer, Diseases, Medical research, Risk factors

## Abstract

Oral epithelial dysplasia (OED) is considered a risk for oral squamous cell carcinoma (OSCC). A meta-analysis estimated a mean malignant transformation rate of 12.1% (95% CI 8.1–17.9). The main target of this study was to define how many OED patients develop OSCC in the hospital district of Southwest Finland. A total of 571 patients diagnosed with OED were identified. Their potential subsequent diagnosis of OSCC was derived from the Finnish Cancer Registry. The risk of OSCC development in OED patients was compared with that of the general population without OED. During a mean follow-up of 5.5 (range 0.1–29.0) years 10.9% of OED patients developed OSCC. OED patients had a 44.7-fold higher risk (95% CI 34.4–56.7) of developing OSCC than the general population. The risk was at its highest within two years of OED diagnosis. OED patients in Southwest Finland have a significantly increased risk of developing OSCC relative to the general population, especially within the first two years of dysplasia diagnosis.

## Introduction

Oral epithelial dysplasia (OED) is defined as an epithelial tissue in which the prevalence of oral squamous cell carcinoma (OSCC) is higher than in its healthy counterpart^1^. OED is a histopathological diagnostic term for a premalignant disorder of oral epithelia, nowadays known as a potentially malignant lesion. However, not all OEDs proceed to OSCC.

OED is traditionally graded by a pathologist in three categories by increasing severity: mild, moderate, and severe^2,3^. Histopathological diagnosis for grading OED is by its nature imprecise, and interobserver agreement in OED grading is poor^4,5^. A newer grading system categorizing OED into two grades, low and high risk, aims to increase objectivity of grading^1^.

The main etiological factors for OED are smoking and alcohol consumption, similarly as with oral carcinoma itself^1^. Clinically, OED typically presents as leukoplakia, erythroplakia, or leukoerythroplakia^4,6^. Oral leukoplakia is a clinical term for a white or pale lesion of the oral mucosa. It is based on visual examination and palpation and should be used only as a clinical term. Defined by the World Health Organization (WHO), leukoplakia is “a white plaque of questionable risk, having excluded (other) known diseases or disorders that carry no increased risk for cancer”^7^. Erythroplakia and leukoerythroplakia have similar definitions based on color and visual appearance^7,8^. A prerequisite for the clinical diagnosis of dysplasia is a biopsy of the lesion to check for OED^1^.

A systematic review and meta-analysis by Iocca et al.^9^ found the annual malignant transformation rate for mild OED to be 1.7% and for severe OED 3.57%. Several studies have reported higher malignant transformation rates among patients with higher OED grades^10–12^. However, some studies have not found a correlation between transformation rate and OED grade^13,14^. In addition, the malignant transformation rates have varied according to geographical region. For example, the malignant transformation rate of severe/carcinoma in situ OED was reported to be 3.2% in the UK^15^ and 50% in Japan^16^.

The risk of malignant transformation does not disappear when the OED lesion is surgically excised, mostly likely because of the field cancerization theory^17–20^. A study by Mehanna et al.^10^ noted, however, that a fully removed OED lesion had a lower malignant transformation rate than those that were incompletely removed. Monitoring OED lesions can enable early detection of malignant transformation. Early detection of OSCC improves the patient’s prognosis^9,21^. The purpose of this study was to determine the malignant transformation rate of OED and the timespan for progression to OSCC in the population of Southwest Finland. This information will improve the follow-up protocols and treatment guidelines of OED.

## Materials and methods

### Cases

All methods in this study were performed in accordance with Finnish guidelines and legislation. The study protocol was approved by the Finnish Institute for Health and Welfare (decision no. THL/1475/5.05.00/2018) and the Institute of Dentisty, University of Turku, Finland as a registry holder. Separate ethics approval was not required because material was derived from a public registry (TENK2009). This study was retrospective and registry based. No patient was contacted during the study. Patients consent was waived by the Finnish Institute for Health and Welfare (decision no. THL/1475/5.05.00/2018).

Patients were retrospectively identified through their OED samples from the Department of Oral Pathology and Oral Radiology, University of Turku between the years 1980 and 2011. Altogether 895 surgical biopsy samples with a histopathological diagnosis of OED were available. The inclusion criteria were that (1) the location of OED was the oral cavity and (2) the final pathological anatomical diagnosis (PAD) was epithelial dysplasia. We excluded (1) cases with no Finnish social security number available in the referral letter (n = 27), (2) cases with previous or simultaneous carcinoma of the head and neck region (n = 36), and (3) cases with simultaneous candidiasis (n = 57). Only the first biopsy sample from each patient was included in this study, resulting in the exclusion of 204 samples. This was done in order to evaluate the malignant transformation process and time from the beginning of the disease. The total number of included patients was 571. OED diagnoses were based on routine histopathological diagnosis. No samples were re-examined. Information on patients and lesions was gathered from the biopsy referral letters and included gender, date of birth, age at diagnosis, histopathologic diagnosis, and date of dysplasia diagnosis.

### Follow-up

Patients were identified by their Finnish social security number. This number was compared with data from the Finnish Cancer Registry (FCR) to determine whether the patients had developed OSCC^22^. ICD codes C00, C02–C06 were considered as OSCC in this study^23^. The FCR, founded in 1952, maintains the national registry of the National Institute for Health and Welfare on all cancer cases in Finland^22^. The FCR also provided the date of OSCC diagnosis, age at time of diagnosis, and ICD-O-3 topographical and morphology codes. The Official Statistics of Finland (OSF), founded in 1865, provided the date of death of patients when applicable^24^. Otherwise, the follow-up ended on December 31, 2017.

### Statistics

Statistical analysis of the data was provided by the FCR. The standardized incidence ratio (SIR) analysis compares the cohort disease incidence with that of the general population^25^. SIR was calculated by taking into account patients’ person-years, age, and gender. The reference population comprised the people living in the region of the hospital district of Southwest Finland. Information included cancer cases and person-years of the reference population. Confidence intervals for SIR are based on the Poisson model. In the calculation, the primary OSCC of each patient was used. All OED patients had a minimum five-year follow-up after their first OED diagnosis.

## Results

### Cases

**Figure 1:**
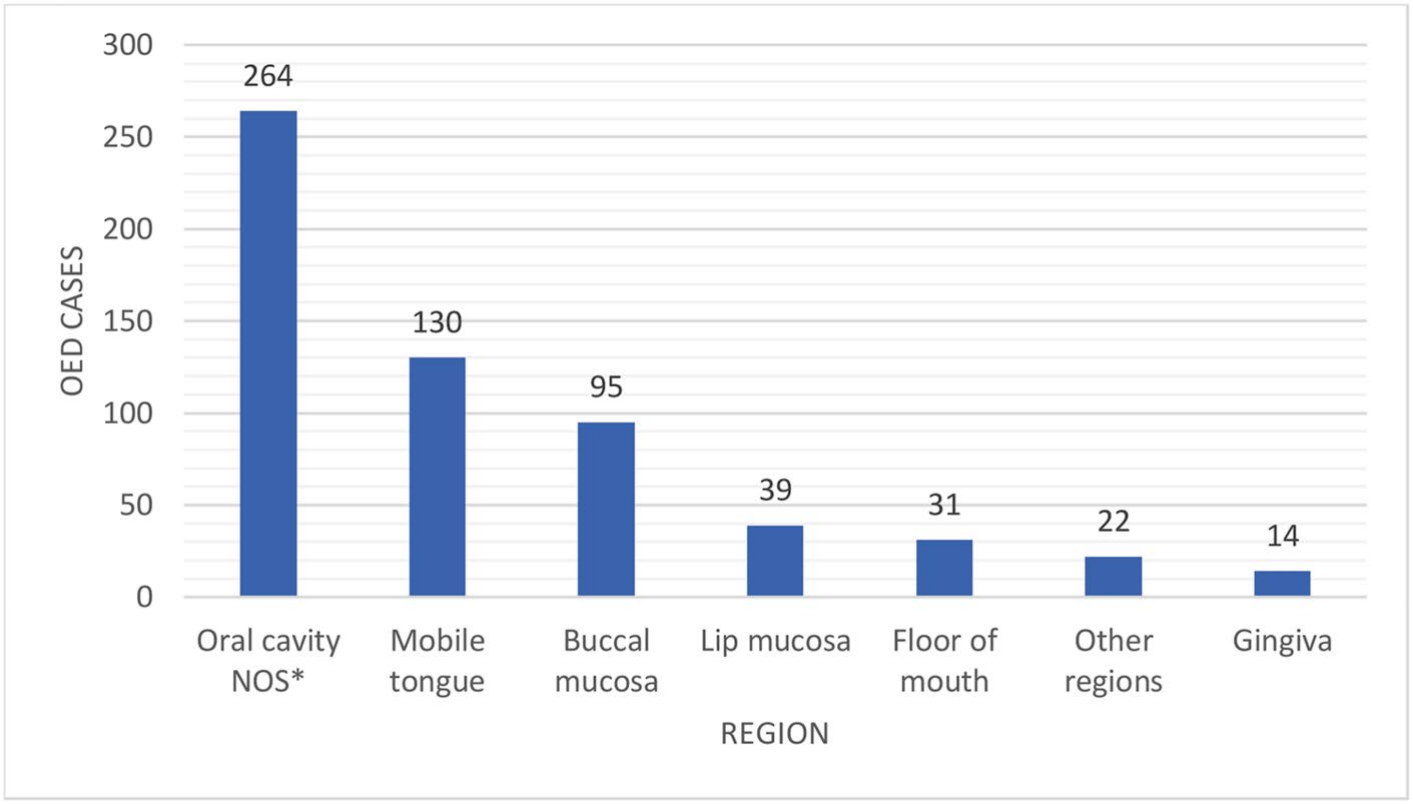
Location of oral epithelial dysplasia (OED) cases in the oral cavity (*NOS not otherwise specified).

Altogether 571 patients with OED were included in this study. The mean age of patients at the time of OED diagnosis was 59.0 (range 15–93) years. Of the patients, 59.2% were women and 40.8% men. OED diagnoses were graded as mild (68.3%), moderate (25.0%), and severe (2.3%). In addition, 25 of the dysplasia diagnoses had no dysplasia graded, mostly from the early years in 1985–1988. Of the 571 OED cases, 307 (53.8%) had information available on the exact biopsy site available. The most common site for OED was the mobile tongue (42.3%) (Fig. 1).

### Malignant transformation

**Figure 2:**
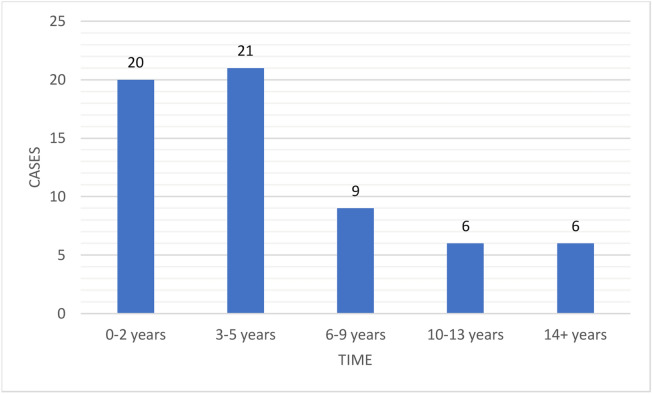
Malignant transformation time from oral epithelial dysplasia to oral squamous cell carcinoma.

**Figure 3:**
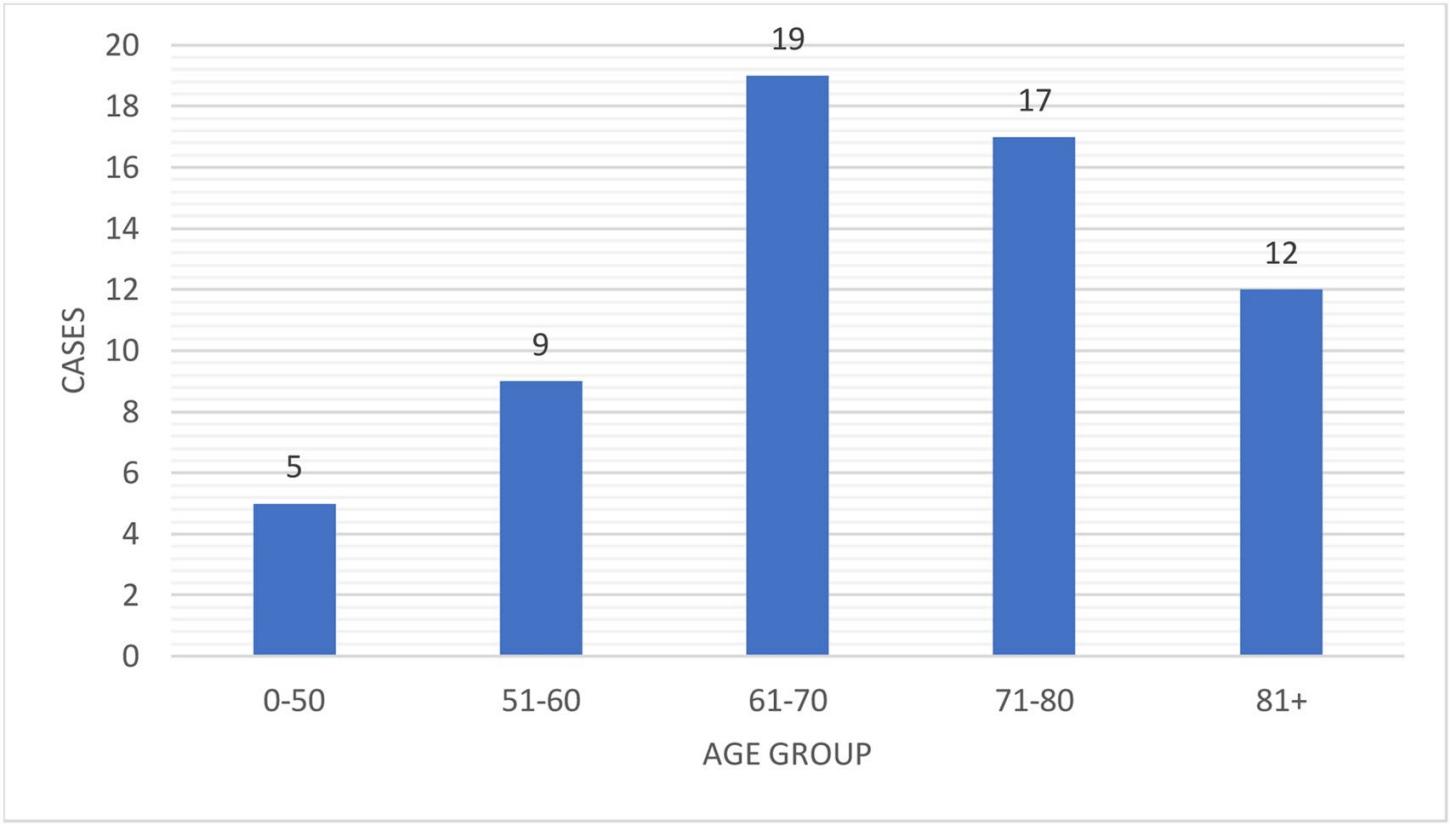
Age distribution of oral squamous cell carcinoma patients at time of diagnosis.

Of the 571 patients, 62 (10.9%) developed OSCC (ICD codes C00.9–C06.9) during follow-up. The timespan for OSCC development varied between 0.1 and 29.0 years. The mean timespan for OSCC development was 5.6 years, with a standard deviation (SD) of 5.9 years (Fig. 2). The mean age of patients at the time of OSCC diagnosis was 68.3 (SD 12.3) years (Fig. 3). Of the 62 patients, 42 were women and 20 men. Four patients had multiple primary OSCC diagnoses during the follow-up period. Of the patients diagnosed with OSCC, 23 (37.1%) developed OSCC at unspecified parts of the tongue, 12 (19.4%) in the border of the tongue, 10 (16.1%) in the floor of the mouth, 9 (14.5%) in the gums, and 8 (12.9%) in other parts of the oral cavity. Of the patients with specific OED information available (n = 307), OSCC developed in the same site as the pre-existing OED in 27 patients (8.8%) and in a different site in 8 patients (2.6%). For 27 patients (8.8%), the OSCC site in relation to the OED site was inconclusive. Considering the OED grades (n = 546), 8.2% of the mild-graded OEDs, 16.1% of the moderate-graded OEDs, and 38.5% of the severe-graded OEDs transformed to OSCC.

### Standardized incidence ratio

**Figure 4:**
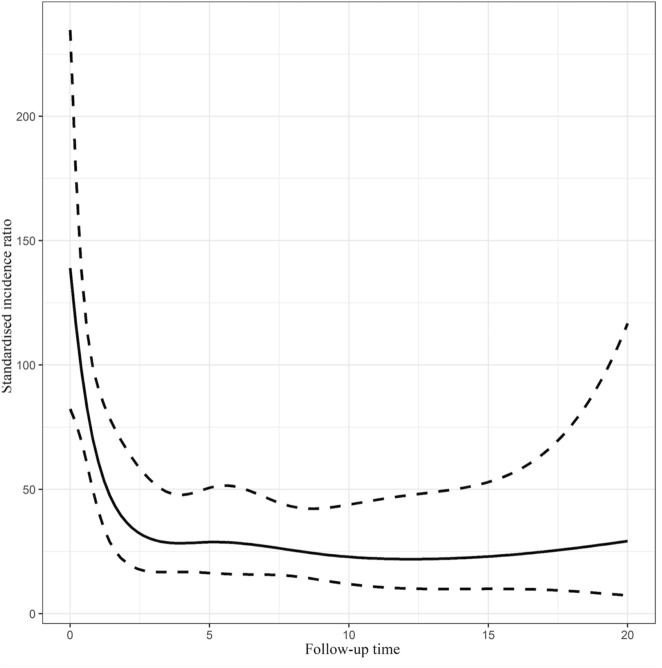
Change in standardised incidence ratio per year of follow-up.

The probability for an OED patient to develop OSCC was compared with that for the general Finnish population of the southwestern region by using SIR. The overall SIR count was 44.7 (95% CI 34.4–56.7), which means a 44.7–fold greater risk for the OED patient to develop OSCC than for the comparable population with no OED. Women`s SIR was 59.7 (95% CI 43.2–79.9) and men`s 29.9 (95% CI 18.9–44.6), indicating that women have a greater risk than men for developing OSCC. SIR varied during the follow-up and was highest within 0–2 years of diagnosis of OED (Fig. 4).

## Discussion

OED is a potentially malignant lesion requiring a life-long follow-up at healthcare services. However, not all OEDs proceed to OSCC. In this retrospective registry study, the overall malignant transformation rate for OEDs in the hospital district of Southwest Finland was 10.9%. This is consistent with the figures reported in the literature. A systematic review and meta-analysis yielded a mean malignant transformation rate of 12.1% (95% CI 8.1–17.9%)^10^. There is considerable heterogeneity of rates between studies, which is most likely explained by different geographical regions, ethnicities, and lifestyles such as tobacco and alcohol use. Unlike other studies, we chose to use the first dysplasia diagnosis instead of the worst biopsy when sequential biopsies were available in order to cover the total timespan before OSCC diagnosis.

The main finding of this study was that the probability of OSCC is significantly higher in patients diagnosed with OED than in the general population in the hospital district of Southwest Finland. The SIR was 44.7, which means that OED-diagnosed patients have 44.7 times greater risk of undergoing malignant transformation than the general population. SIR values varied from 34.4 to 56.7; even at the lower end of the scale, the risk for OSCC is high. SIR for women was 59.7 and for men 29.9, highlighting the greater risk for women of undergoing malignant transformation. According to our knowledge, no other SIR analysis for OED malignant transformation has been performed to date.

The malignant transformation rate varied during the follow-up (Fig. 2). The risk was highest during the first two years after OED diagnosis as reported earlier by Silverman et al.^11^. In the literature, the risk for malignant transformation has been considered most often the highest within the first 5 years of diagnosis^13,26^. A meta-analysis by Mehanna et al.^10^ found that the mean time to malignant transformation of OED was 4.3 years. This information aids clinicians in planning follow-up schema.

Several studies—including ours—have evaluated how OED grading correlates with OSCC transformation. Widely varying results have emerged, from no correlation at all to a clear correlation^11,13,14^. In the meta-analysis by Mehanna et al.^10^, the malignant transformation rate for mild to moderate OED was 10.3% and for severe OED 24.1%. In our study, 8.2% of mild-graded OEDs, 16.1% of moderate-graded OEDs, and 38.5% of severe-graded OEDs transformed to OSCC, showing that severity of OED entailed more risk (Table 1). One explanation for the variable results may be that histopathological diagnosis of OED is subjective, especially the grading^4,5^. WHO has tried to resolve this issue by suggesting that OED be graded into only two categories, i.e. low-risk and high-risk grades^1^. In our study, we did not re-evaluate the histopathological diagnoses, relying instead on the registry-based diagnoses to represent the real life situation. Similarly, we did not include the diagnosis of differentiated dysplasia since it was not recognized in 1980–2011.

**Table 1:** Transformation of oral epithelial dysplasia (OED) to oral squamous cell carcinoma (OSCC).

OED grade (n)	OSCC cases	Transformation rate
Mild (390)	32	8.1
Moderate (143)	23	16.1
Severe (13)	5	38.5

The risk for OSCC was higher among women. Malignant transformation rate for men was 8.6% and for women 12.4%, and the SIR was significantly higher for women. Other studies have also reported a higher risk for women of developing OSCC^13^. The exact reason for this sex difference is unknown. An explanation has been sought in smaller body size, hormonal factors, lifestyle/habits, viral infections, and genetics^27–29^.

Location of the OED lesion could have an influence on the OED transformation rate. Sites with the highest risk of malignant transformation have been reported to be the lateral sides of the tongue, the floor of the mouth, and the buccal mucosa^26^. In our study, the most common site for OSCC was indeed the mobile tongue. Of tongue OSCC, 34.3% were located on the lateral sides of the tongue. Therefore, the relatively high transformation rate could at least partly be explained by lesions in risk areas.

The main strength of this study is the relatively high number of OED cases compared with other current studies. A registry-based approach is another strength. The OSF and FCR registries have excellent coverage^30,31^. The OSF has almost 100% coverage of death statistics in Finland and the FCR a coverage and accuracy of over 98% of all cancers in Finland. However, because this was a retrospective and registry-based study, weaknesses include having no correlation with medical history, risk factors such as smoking and alcohol consumption, social history, or details or treatment of the lesion. Smoking and drinking habits are recognized to be the main etiological factors for OED. As the original OED diagnosis was used, the variability of reporting among several pathologists over the period of 1980–2011 has an impact on the findings. While this is a limitation of the study, the original OED diagnosis was chosen in order to represent the real-life situation, in accord with an Australian study^32^. In addition, histopathological reporting of OED is known to be subjective^32,33^.

In conclusion, we have shown that OED patients have 44.7 times higher risk of developing OSCC than their healthy counterparts. The greatest risk is within two years of OED diagnosis. Women and more severe OED grades also have a larger risk. This information will aid clinicians in determining individual follow-up schemes for early and active surveillance of OED patients.

## Supplementary Information

Supplementary Information.

## Data Availability

All data generated or analyzed during this study are included in this published article (and its Supplementary Information files).
